# A novel approach with a fuzzy sliding mode proportional integral control algorithm tuned by fuzzy method (FSMPIF)

**DOI:** 10.1038/s41598-023-34455-7

**Published:** 2023-05-05

**Authors:** Tuan Anh Nguyen

**Affiliations:** Thuyloi University, Hanoi, Vietnam

**Keywords:** Mechanical engineering, Electrical and electronic engineering

## Abstract

An automobile's vibration can be caused by stimulation from the road's surface. The change in displacement and acceleration values of the sprung mass is used to evaluate the automobile's vibration. Utilizing an active suspension system is recommended in order to attain an increased level of ride comfort. This article presents a novel strategy for regulating the operation of an active suspension system that has been put up for consideration. The PI (Proportional Integral) algorithm, the SMC (Sliding Mode Control) algorithm, and the Fuzzy algorithm served as the basis for developing the FSMPIF algorithm. The signal generated by the SMC algorithm is what is used as the input for the Fuzzy algorithm. In addition, the settings of the PI controller are modified with the help of yet another Fuzzy algorithm. These two Fuzzy methods operate independently from one another and in a setting that is wholly distinct from one another. This algorithm was created in a wholly original and novel way. Using a numerical modelling technique, the vibration of automobiles is investigated with a particular emphasis on two distinct usage situations. In each case, a comparison is made between four different circumstances. Once the FSMPIF method is implemented, the results of the simulation process have demonstrated that the values of displacement and acceleration of the sprung mass are significantly decreased. This was determined by looking at the values before and after implementing the new algorithm. In the first case, these figures do not surpass a difference of 2.55% compared to automobiles that use passive suspension systems. The second case sees these figures falling short of 12.59% in total. As a direct result, the automobile's steadiness and level of comfort have been significantly improved.

## Introduction

The automobile's comfort and steadiness are crucial factors. It can impact the comfort of the vehicle's passengers. The suspension system guarantees the proper level of ride comfort^1^. Typically, the suspension system is between the vehicle's body and the wheel. The components above a suspension system are known as the sprung mass (vehicle body). The components underneath a suspension system are referred to as unsprung mass^2^. A suspension system's primary components are a shock damper, lever arms (upper or lower lever arm), and springs (coil spring, leaf spring)^3^. According to certain studies, the anti-roll bar is also a suspension system component^4,5^. Compared to other systems, the suspension system's construction is relatively complex.

Uneven road surfaces are the primary source of automobile vibration, according to Zuraulis et al.^6^. Several more variables can also contribute to variations. However, the impact of these variables is negligible. Wheel vibrations are transferred to the car body via the suspension system. The suspension system will regulate these vibrations. In addition, the suspension system will decrease the vibration energy. When analyzing the vibration of a vehicle, several factors are considered, but displacement and acceleration values of the sprung mass are vital factors. These two markers have been utilized in much earlier research^7,8^. The displacement and acceleration of a vehicle body can be determined by simulation or experiment. Only the highest vehicle body displacement and acceleration values should be considered for discontinuous vibrations. The average and maximum values of the two parameters above may be employed for continuous vibrations. RMS critical allows for calculating mean values^9–11^.

The performance of the passive suspension system (mechanical suspension system) is poor. It does not meet the requirements for smoothness for substantial frequencies and continuous volume excitations. Instead of this, mechatronics suspension system solutions should be utilized. Zhang et al. presented the pneumatic spring suspension^12^. This system utilizes balloons with completely automated control systems. These pneumatic balloons are variable-stiffness pneumatic springs^13^. The hardness of pneumatic springs may be altered by adjusting the pressure within pneumatic balloons. This was emphasized by Geng et al.^14^. When a vehicle is equipped with a pneumatic suspension system, its ride quality is good. However, this type is rather expensive. The use of electromagnetic absorbers to replace traditional absorbers often described as a “semi-active suspension system,” is another technology presented by Oh et al.^15^. According to Basargan et al., the current within the damper will alter the arrangement of the metal particles in its vicinity. Consequently, the damping stiffness is continually variable^16^. This kind is simpler and less expensive. Their efficacy, however, is typical. To better manage the automobile's vibrations, an extra actuator is required to upgrade the suspension system. Based on this approach, an active suspension system was implemented^17^. The active suspension system incorporates a hydraulic actuator. This actuator may apply force on the vehicle's mass from two sides. Consequently, its performance will improve. Nevertheless, the suspension system's construction will become more complex. Additionally, active suspension is more expensive than a semi-active suspension system.

Recently, several publications concerning control for suspension systems have been published. Nguyen proposed i^18^ employing the double combined PID (Proportional Integral Derivative) controller for the vehicle's quarter-dynamics model. This integrated double controller comprises two separate controllers. Each component controller regulates a distinct parameter. The PID controller's parameters K_P_, K_I_, and K_D_, must be chosen suitably. If a FOPID (Fractional Order Proportional Integral Derivative) controller is used in place of a PID controller, the number of variables will double^19^. Han et al.^20^ developed a Fuzzy method to modify these settings. According to Mahmoodabadi and Nejadkourki's demonstration^21^, the value of these three factors may be altered continually. In addition, intelligence algorithms have been employed to optimize the PID controller settings^22–24^. For systems with multiple objects, either LQR (Linear Quadratic Regulator) or LQG (Linear Quadratic Gaussian) control algorithms are preferable^25^. By reducing the cost function, this approach will assist in optimizing automobile vibration^26^. Frequently, the preceding techniques are used to operate linear systems. The SMC method must be utilized for nonlinear systems. According to Azizi and Mobki, the objects will slide over the surface. The object then advances toward the location of equilibrium^27^. According to Nguyen, a sliding surface is a complicated function dependent on the controller's error signal^28^. The error signal is evaluated using the derivative of a high order. In order to ease the issue, it is essential to linearize a hydraulic actuator. This information was provided by Nguyen et al.^11^. Combining the SMC and Fuzzy techniques will improve its performance^29^. This has been demonstrated by Chen et al. in^30^ when they used a combination of SMC and Fuzzy algorithms for the nonlinear system. Besides, the Fuzzy adaptive algorithms also help better observe the system's error state^31^. Many other intelligent control algorithms have also been applied to the suspension controller. In^32^, Liu et al. introduced the ANN (Adaptive Neural Network) algorithm for active suspension with constraints related to vehicle speed and displacement. The parameters of the controllers for the suspension can be optimally selected through methods such as GA (Genetic algorithm)^33^ or PSO (Particle Swarm Optimization)^34^. Some techniques that use artificial intelligence to design the suspension controller have also been applied to heavy trucks^35^. In addition, several suspension system control methods are highly efficient^36,37^.

In order to meet the specifications for the automobile's ride comfort, it is crucial to regulate the suspension system's operation. The authors offer an original control algorithm, FSMPIF, in this work, based on four distinct perspectives. Besides, the controller design procedure is described in the article's content. In addition, a numerical simulation approach is used to analyze the vibration of the automobile. This article consists of four sections. In the Introduction section, some concepts and literature reviews are pointed out. In the Models section, the authors will explain the process of establishing a vehicle dynamics model and a control algorithm. The calculation and simulation process are done in the Results and Discussions section next. Finally, some comments will be indicated in the Conclusions section. In the following sections of the article, specific details are offered.

## Models

**Figure 1:**
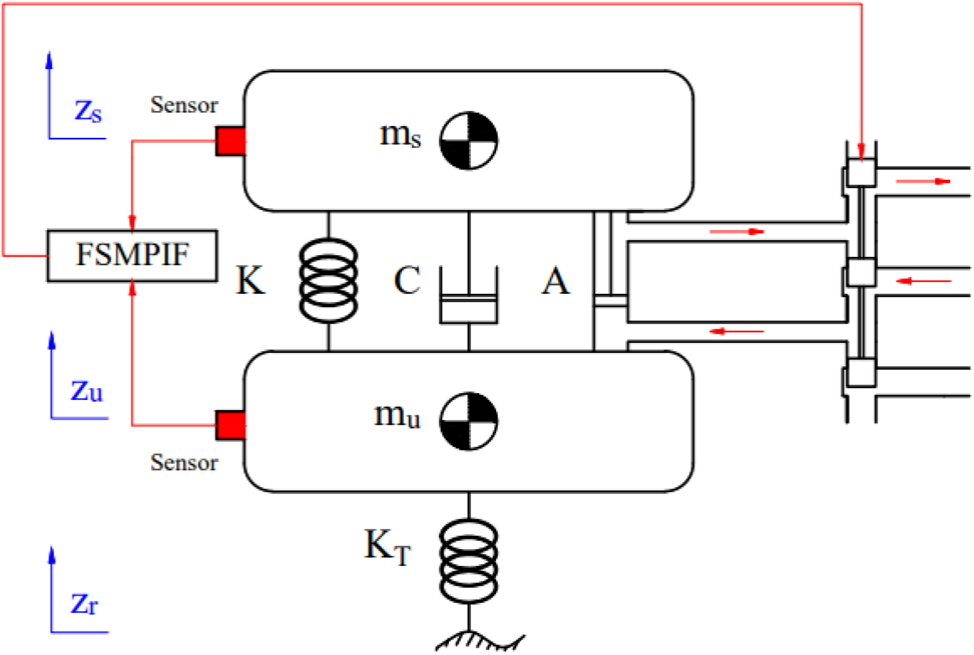
A quarter-dynamics model.

Initially, developing a dynamics model of the vehicle's vibrations is necessary. This research used a quarter-dynamics model with two masses; *m*_*s*_ will produce the vertical displacement *z*_*s*_, whereas *m*_*u*_ will do the vertical displacement *z*_*u*_ (Fig. 1).

The differential equations describing vehicle vibrations are listed as follows:

1 $$ F_{ims} - F_{C} - F_{K} - F_{A} = 0 $$

2 $$ F_{imu} + F_{C} + F_{K} + F_{A} - F_{KT} = 0 $$

where:

3 $$ F_{ims} = m_{s} \ddot{z}_{s} $$

4 $$ F_{imu} = m_{u} \ddot{z}_{u} $$

5 $$ F_{K} = K\left( {z_{u} - z_{s} } \right) $$

6 $$ F_{KT} = K_{T} \left( {z_{r} - z_{u} } \right) $$

7 $$ F_{C} = C\left( {\dot{z}_{u} - \dot{z}_{s} } \right) $$

8 $$ F_{A} = \rho_{1} \int\limits_{0}^{t} {\left( {u\left( \tau \right) - \rho_{2} F_{Ac} } \right)d\tau } + \rho_{3} \left( {z_{u} - z_{s} } \right) $$

Substituting Eqs. (3) to (8) into Eqs. (1) and (2) produces:

9 $$ m_{s} \ddot{z}_{s} - C\left( {\dot{z}_{u} - \dot{z}_{s} } \right) - K\left( {z_{u} - z_{s} } \right) - \rho_{1} \int\limits_{0}^{t} {\left( {u\left( \tau \right) - \rho_{2} F_{Ac} } \right)d\tau } + \rho_{3} \left( {z_{u} - z_{s} } \right) = 0 $$

10 $$ m_{u} \ddot{z}_{u} + C\left( {\dot{z}_{u} - \dot{z}_{s} } \right) + K\left( {z_{u} - z_{s} } \right) + \rho_{1} \int\limits_{0}^{t} {\left( {u\left( \tau \right) - \rho_{2} F_{Ac} } \right)d\tau } + \rho_{3} \left( {z_{u} - z_{s} } \right) - K_{T} \left( {z_{r} - z_{u} } \right) = 0 $$

The control signal of the actuator, *u(t)*, is determined by its controller. A completely innovative control algorithm named FSMPIF is proposed. This algorithm is developed with the following perspectives in mind:

Firstly, the PI algorithm provides a more stable response, whereas the Fuzzy algorithm is more adaptable. Both of these components must concurrently exist in the control signal. Therefore, these two algorithms are required to combine an ultimate control signal.

Secondly, the PI algorithm settings must be adjusted appropriately. These values must be modified to accommodate the pavement's excitation signals. Consequently, they must be controlled by a fuzzy system. The vibration of the vehicle body is the input signal for the first fuzzy controller.

Thirdly, because the vehicle vibration is nonlinear, it is essential to design a nonlinear control algorithm to fulfill the system's stability requirements. The SMC algorithm is appropriate for this function. The output signal of the SMC technique will serve as the input signal for the second fuzzy controller described in the first point.

Fourthly, the second fuzzy controller is a crucial component of the integrated controller. Consequently, the signal of the second Fuzzy controller will consist of three components: the output signal of the previously described SMC algorithm, the error signal of the PI algorithm, and the vibration signal of the vehicle body.

**Figure 2:**
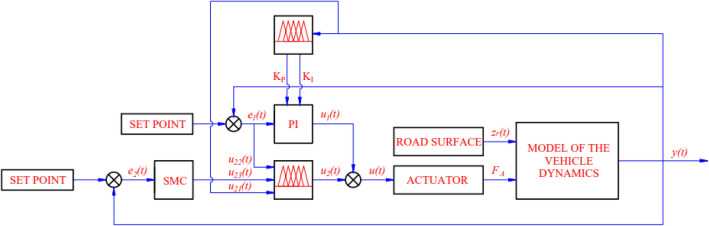
Control system.

On the basis of the considerations above, the Fuzzy Sliding Mode Proportional Integral tuned by Fuzzy (FSMPIF) technique was suggested. This algorithm satisfies all system stability criteria. Figure 2 displays the system's schematic.

Vehicle comfort can be measured through values related to car oscillations, such as displacement and acceleration of the sprung mass. These values are measured directly by the sensors fitted on the car. The result obtained from the sensor is the feedback signal of the system (Fig. 2). When evaluating ride comfort, we often consider the average, RMS, or maximum value.

Synthesis of the final control signals *u(t)* from the two-component signals *u*_*1*_*(t)* and *u*_*2*_*(t)*.

11 $$ u\left( t \right) = u_{1} \left( t \right) + u_{2} \left( t \right) $$

The first element signal, *u*_*1*_*(t)*, is the PI controller's output signal.

12 $$ u_{1} \left( t \right) = K_{P} e\left( t \right) + K_{I} \int\limits_{0}^{t} {e\left( \tau \right)d\tau } $$

13 $$ e\left( t \right) = y_{s} \left( t \right) - y\left( t \right) $$

where: *e(t)*: the error signal of the PI controller, *x*_*s*_*(t)*: setpoint signal, *x(t)*: output signal.

This setpoint signal should be zero so that the vehicle body vibrates as little as possible. It implies:

14 $$ e\left( t \right) = - \ddot{z}_{s} $$

**Figure 3:**
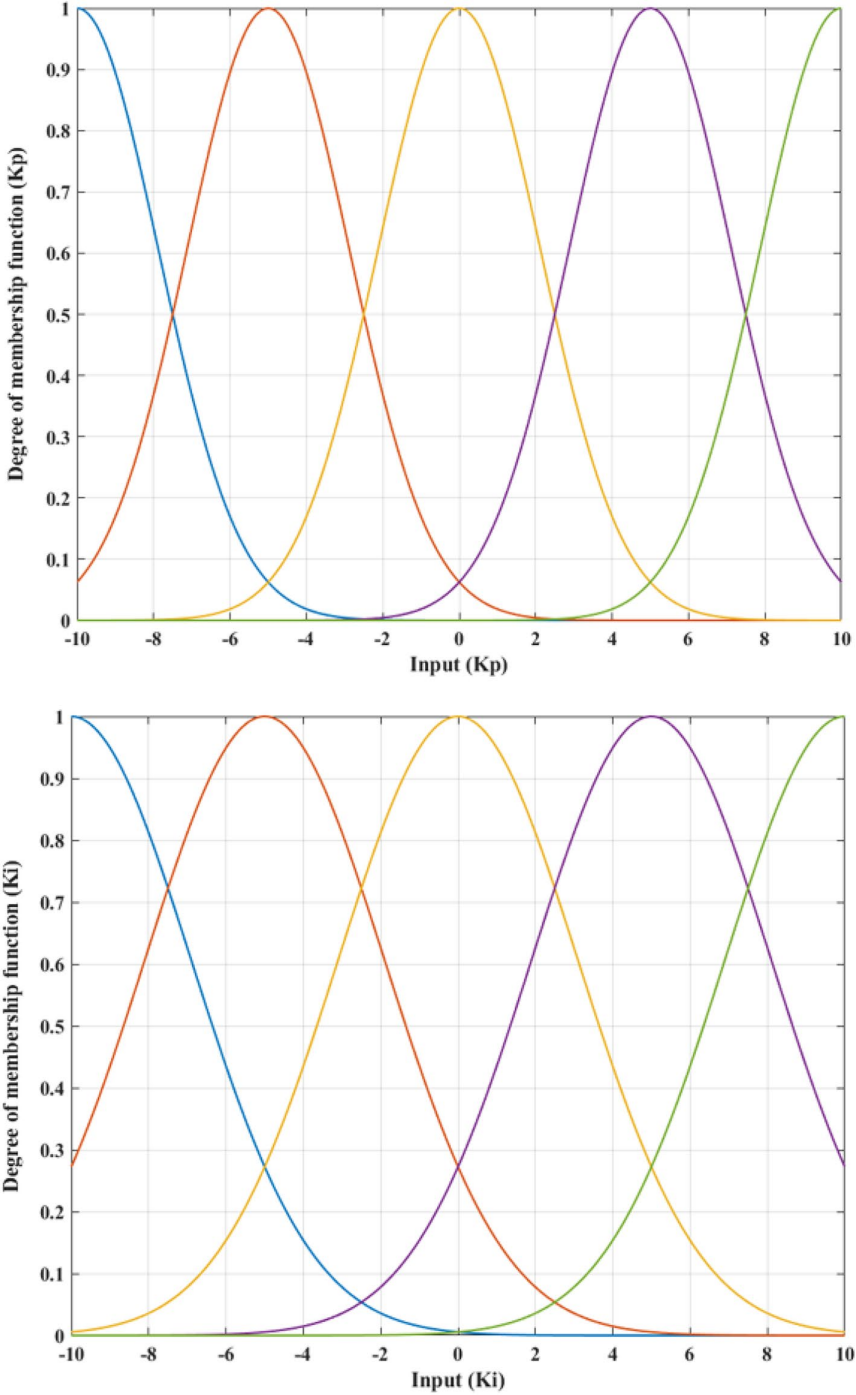
Membership functions of the first Fuzzy controller.

Including the first viewpoint, the PI controller settings must be continually adjusted to fulfill the system's requirements. Therefore, tuning these settings with a Fuzzy system is a viable option. This is the first controller for Fuzzy. This controller's input is the sprung mass displacement value. Figure 3 depicts the membership function of this controller. This function is developed from the perspective of the author. A control signal will be transmitted as soon as the vehicle's body vibrates. Equation (12) can also be expressed as:

15 $$ u_{1} \left( t \right) = - defuzz_{{K_{P} }} \left( {c_{p} z_{s} } \right)\ddot{z}_{s} - defuzz_{{K_{I} }} \left( {c_{i} z_{s} } \right)\int\limits_{0}^{t} {\ddot{z}_{s} \left( \tau \right)d\tau } $$

The second Fuzzy controller's output signal is the integrated controller's second component signal, *u*_*2*_*(t)*, also known as the central controller. The input signals for this controller are *u*_*21*_*(t)*, *u*_*22*_*(t)*, and *u*_*23*_*(t)*.

16 $$ u_{2} \left( t \right) = defuzz\left( {u_{21} \left( t \right) + u_{22} \left( t \right) + u_{23} \left( t \right)} \right) $$

The initial input signal, *u*_*21*_*(t)*, represents the vehicle body displacement. The PI controller error signal is the second input signal, *u*_*22*_*(t)*. It is multiplying a gain factor (*k*_*gf*_) by this signal.

17 $$ u_{21} \left( t \right) = k_{gf} z_{s} \left( t \right) $$

18 $$ u_{22} \left( t \right) = e\left( t \right) = - \ddot{z}_{s} $$

The SMC controller's output signal is the last input signal *u*_*23*_*(t)*. An SMC controller is an integral part of the integrated controller.

Consider a nonlinear control object with *u(t)* as an input signal and *y(t)* as an output signal. A function determined by the component derivation signals and the input signals is referred to as the *nth* derivative of the output signal.

19 $$ y^{\left( n \right)} \left( t \right) = f\left( {y,\dot{y},\ddot{y},...,y^{{\left( {n - 1} \right)}} } \right) + u\left( t \right) $$

In this scenario, it is assumed that the function *f(y)* is limited and subject to uncertainty, i.e.

20 $$ ||f\left( {y\left( x \right)} \right)|| < \delta < \infty $$

Let the following be the values of the model's state variables:

21 $$ \begin{aligned} & x_{1} = y \hfill \\ & x_{2} = \dot{y} \hfill \\ & ... \hfill \\ & x_{n} = y^{{\left( {n - 1} \right)}} \hfill \\ \end{aligned} $$

The object's model is returned as a system of state equations as follows:

22 $$ \left\{ \begin{aligned} & \dot{x}_{i} = x_{i + 1} \hfill \\ & \dot{x}_{n} = f\left( {\left[ \begin{gathered} x_{1} \hfill \\ x_{2} \hfill \\ ... \hfill \\ x_{n} \hfill \\ \end{gathered} \right]} \right) + u\left( t \right) \hfill \\ & y = x_{1} \hfill \\ \end{aligned} \right. $$

Assuming the setpoint signal is zero, condition (21) guarantees the existence of a model-in signal-response controller whenever the nonlinear object (22) is constrained. In this case, the command signal can be restated as:

23 $$ u\left( t \right) = \left( {k + \delta } \right)sgn\left( s \right) $$

where: *s(e)*: sliding surface (when *s(e)* = 0), *e(t)*: error signal.

24 $$ s\left( e \right) = b_{0} e\left( t \right) + b_{1} \dot{e}\left( t \right) + b_{2} \ddot{e}\left( t \right) + \cdots + b_{n - 2} e^{{\left( {n - 2} \right)}} \left( t \right) + e^{{\left( {n - 1} \right)}} \left( t \right) $$

25 $$ e\left( t \right) = h\left( t \right) - y\left( t \right) = - y\left( t \right) $$

The slip surface's bi coefficients must be set correctly, so that (26) is a Hurwitz polynomial. When this condition is fulfilled, state variables return to zero after a specific time (27).

26 $$ p\left( \gamma \right) = b_{0} + b_{1} \gamma^{1} + b_{2} \gamma^{2} + \cdots + b_{n - 2} \gamma^{n - 2} + \gamma^{n - 1} $$

27 $$ \mathop {\lim }\limits_{t \to \infty } \underline {x} \left( t \right) = \underline {0} $$

Due to:

28 $$ \left\{ \begin{aligned} & e = - x_{1} \hfill \\ & x_{i} = - e^{{\left( {i - 1} \right)}} \hfill \\ \end{aligned} \right. $$

As a result, the Eq. (27) may be written as follows:

29 $$ \mathop {\lim }\limits_{T < t \to \infty } e^{\left( i \right)} = \underline {0} $$

where: *T* is a finite time point.

If the equation *s(e)* = 0 includes coefficients bi that fulfill the Hurwitz polynomial (26) condition, the sliding surface *s(e)* tends to zero, i.e.

30 $$ s\left( e \right)\dot{s} < 0 $$

The sliding condition (sliding surface) of the controller is defined by Eq. (30). We get the following from (22), (24), and (30):

31 $$ \dot{s}\left( e \right) = \sum\limits_{i = 0}^{n - 1} {b_{i} e^{{\left( {i + 1} \right)}} } = - \sum\limits_{i = 0}^{n - 2} {b_{i} x_{i + 2} } - \dot{x}_{n} = - \sum\limits_{i = 0}^{n - 2} {b_{i} x_{i + 2} } - f\left( {\underline {x} } \right) - u(t) $$

If *s(e)* is less than zero, the value of (31) is positive; otherwise, it is negative. The control signal *u(t)* may be rewritten as follows by combining (20) and (31):

32 $$ u\left( t \right) = \left\{ \begin{gathered} < - \sum\limits_{i = 0}^{n - 2} {b_{i} x_{i + 2} } - \delta ;s\left( e \right) < 0 \hfill \\ > \sum\limits_{i = 0}^{n - 2} {b_{i} x_{i + 2} } + \delta ;s\left( e \right) > 0 \hfill \\ \end{gathered} \right. $$

The control signal *u(t)*, as given by Eq. (32), is independent of (22). As a result, it is regarded as a reliable controller. If condition (20) is not met, an upper limit of the function *f(y(x))* must be defined, i.e.

33 $$ \left| {f\left( {\underline {x} } \right)} \right| < \left| {g\left( {\underline {x} } \right)} \right|,\forall x $$

Then the condition becomes:

34 $$ u\left( t \right) = \left\{ \begin{gathered} < - \sum\limits_{i = 0}^{n - 2} {b_{i} x_{i + 2} } - g\left( {\underline {x} } \right);s\left( e \right) < 0 \hfill \\ > \sum\limits_{i = 0}^{n - 2} {b_{i} x_{i + 2} } + g\left( {\underline {x} } \right);s\left( e \right) > 0 \hfill \\ \end{gathered} \right. $$

However, traditional SMC control algorithms still often cause the "chattering" phenomenon mentioned in^38^ by Slotine and Li.

The procedure for designing an SMC controller is described in^28^. According to^9^, the SMC controller's output signal may be written as follows:

35 $$ u_{23} \left( t \right) = \frac{{\chi m_{s} m_{u} }}{{K_{T} \rho_{1} }}\left[ { - \sum\limits_{i = 1}^{5} {b_{i} x_{i} \left( t \right)} + \sum\limits_{i = 1}^{4} {p_{i} \left( { - \ddot{z}_{s} } \right)^{{\left( {4 - i} \right)}} } + Rsgn\left( {\sum\limits_{i = 0}^{4} {p_{i} \left( { - \ddot{z}_{s} } \right)^{{\left( {4 - i} \right)}} } } \right)} \right] $$

The second component signal is a complicated function of the vehicle body vibration signal when Eqs. (16), (17), (18), and (19) are combined.

36 $$ u_{2} \left( t \right) = defuzz\left\{ {k_{gf} z_{s} \left( t \right) - \ddot{z}_{s} \left( t \right) + \frac{{\chi m_{s} m_{u} }}{{K_{T} \rho_{1} }}\left[ { - \sum\limits_{i = 1}^{5} {b_{i} x_{i} \left( t \right)} + \sum\limits_{i = 1}^{4} {p_{i} \left( { - \ddot{z}_{s} } \right)^{{\left( {4 - i} \right)}} } + Rsgn\left( {\sum\limits_{i = 0}^{4} {p_{i} \left( { - \ddot{z}_{s} } \right)^{{\left( {4 - i} \right)}} } } \right)} \right]} \right\} $$

where: *χ* is the ratio coefficient between the inertial forces. This should be referenced in^28^.

**Figure 4:**
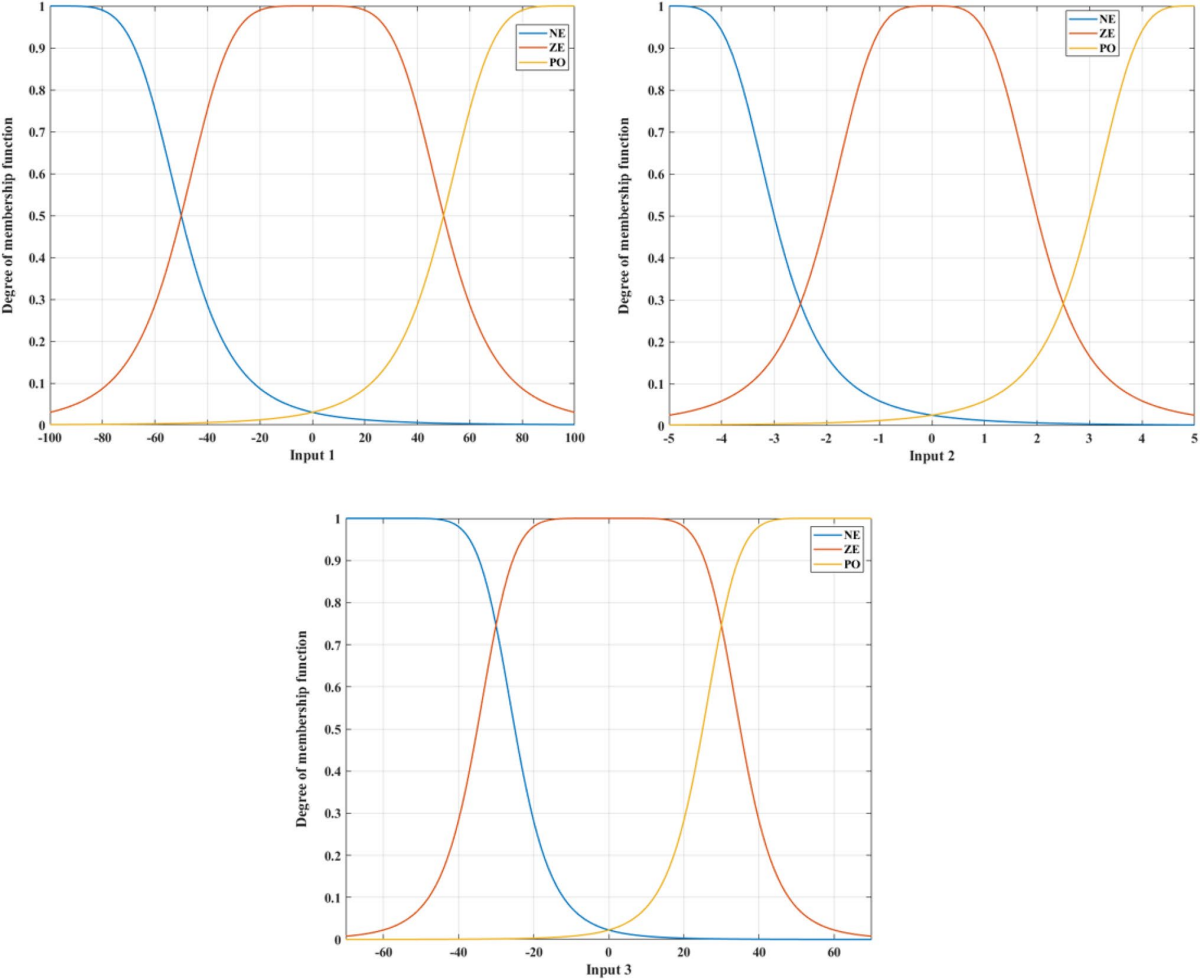
Membership functions of the second Fuzzy controller.

**Figure 5:**
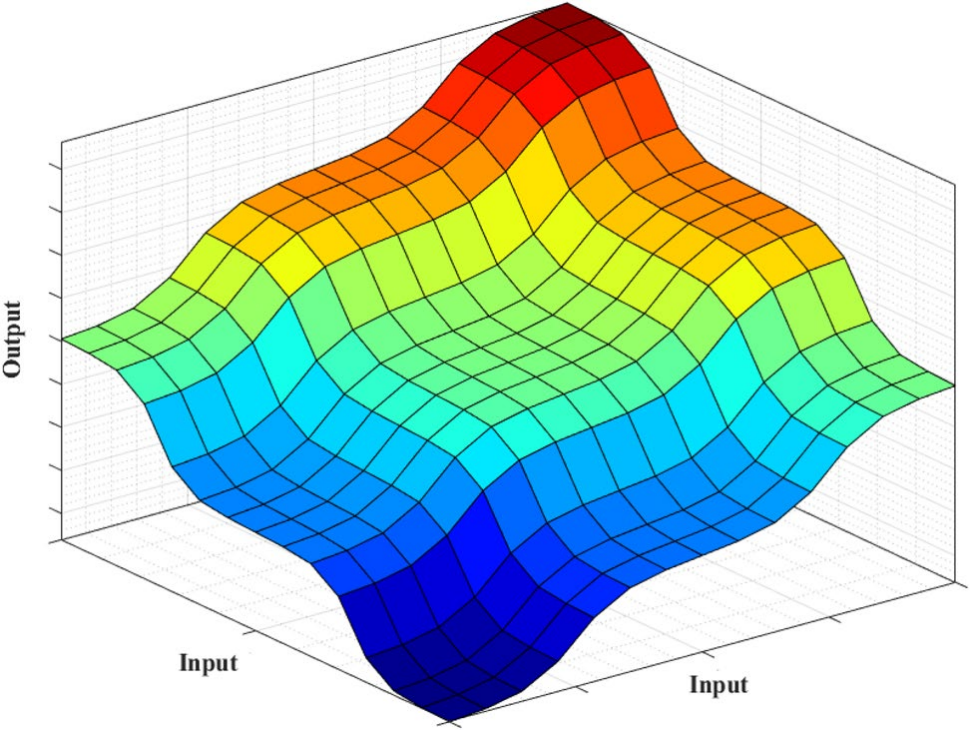
Fuzzy surface.

Figure 4 displays the membership function of this approach. The defuzzification procedure is carried out following the fuzzy rules specified in Table 1 and Fig. 5.

**Table 1:** Fuzzy rules.

Input 1	Input 2	Input 3	Output	Input 1	Input 2	Input 3	Output
NE	NE	NE	BN	ZE	ZE	PO	SP
NE	NE	ZE	BN	ZE	PO	NE	ZE
NE	NE	PO	SN	ZE	PO	ZE	SP
NE	ZE	NE	BN	ZE	PO	PO	BP
NE	ZE	ZE	SN	PO	NE	NE	SN
NE	ZE	PO	ZE	PO	NE	ZE	ZE
NE	PO	NE	SN	PO	NE	PO	SP
NE	PO	ZE	ZE	PO	ZE	NE	ZE
ZE	PO	PO	SP	PO	ZE	ZE	SP
ZE	NE	NE	BN	PO	ZE	PO	BP
ZE	NE	ZE	SN	PO	PO	NE	SP
ZE	NE	PO	ZE	PO	PO	ZE	BP
ZE	ZE	NE	SN	PO	PO	PO	BP
ZE	ZE	ZE	ZE	–	–	–	–

## Results and discussions

### Condition of the simulation process

**Figure 6:**
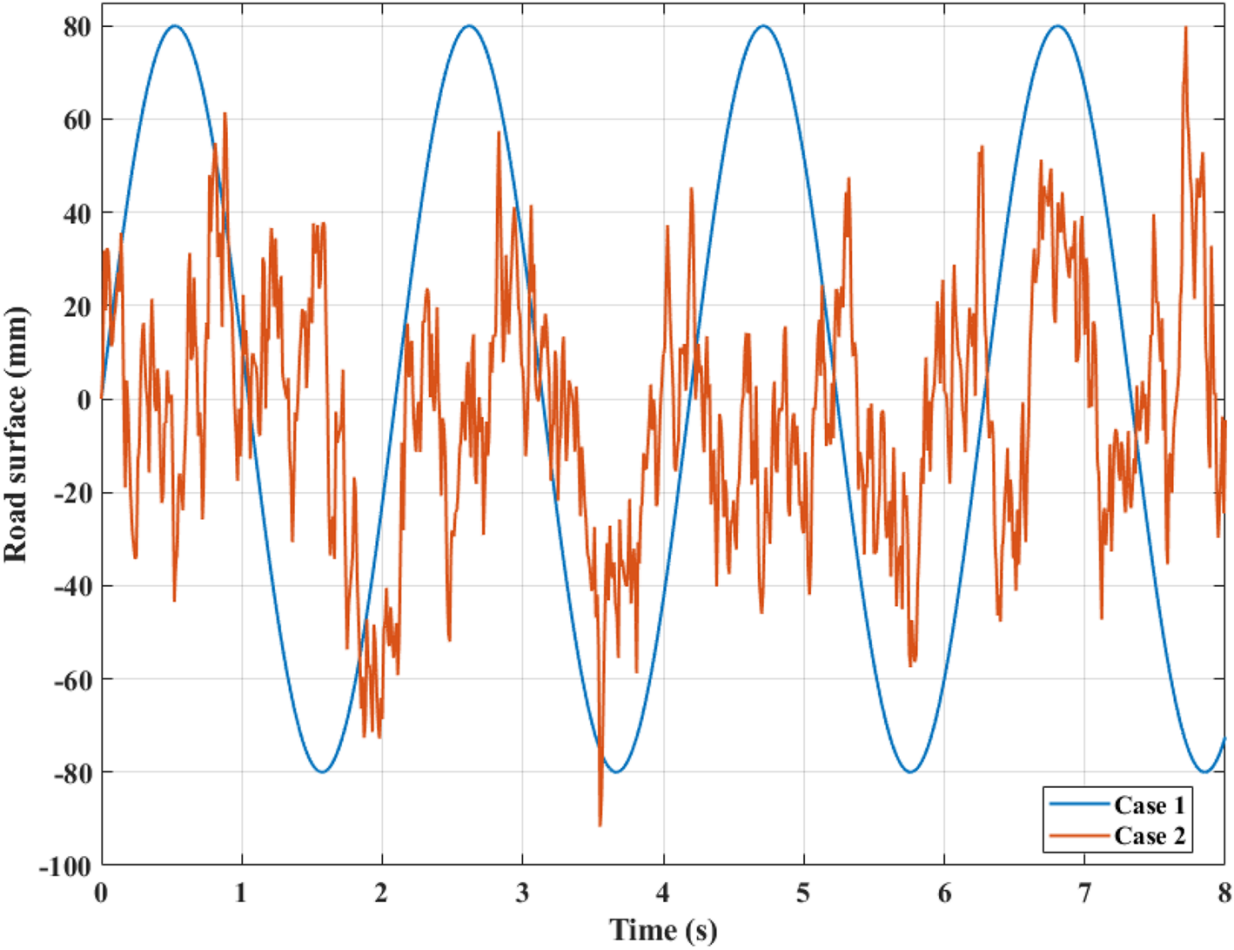
Roughness on the road.

This study uses numerical simulation as its approach. This approach utilizes the MATLAB-Simulink ecosystem. The specs of the vehicle are listed in Table 2. These parameters are taken from the CARSIM^®^ application and slightly modified. Two case studies were conducted corresponding to two forms of road surface excitation (Fig. 6). In each scenario, the vibration of a vehicle will be evaluated under four conditions: passive suspension, PID, SMC, and FSMPIF. With road roughness as the input excitation signal, the system's output signal is the vehicle body displacement and acceleration. Maximum and average (RMS-calculated) outcomes for each condition will be compared.

**Table 2:** Specifications of the vehicle.

Symbol	Description	Value	Unit
m_s_	Sprung mass	465	kg
m_u_	Unsprung mass	51	kg
C	Damper coefficient	3190	Ns/m
K	Spring coefficient	36,500	N/m
K_T_	Tire coefficient	177,000	N/m
ρ_1_	Actuator coefficient	539,561	N^3/2^/kg^1/2^m^1/2^ V
ρ_2_	Actuator coefficient	1	1/s
ρ_3_	Actuator coefficient	5,512,500	N/m

### Results of the simulation process

#### Case 1

**Figure 7:**
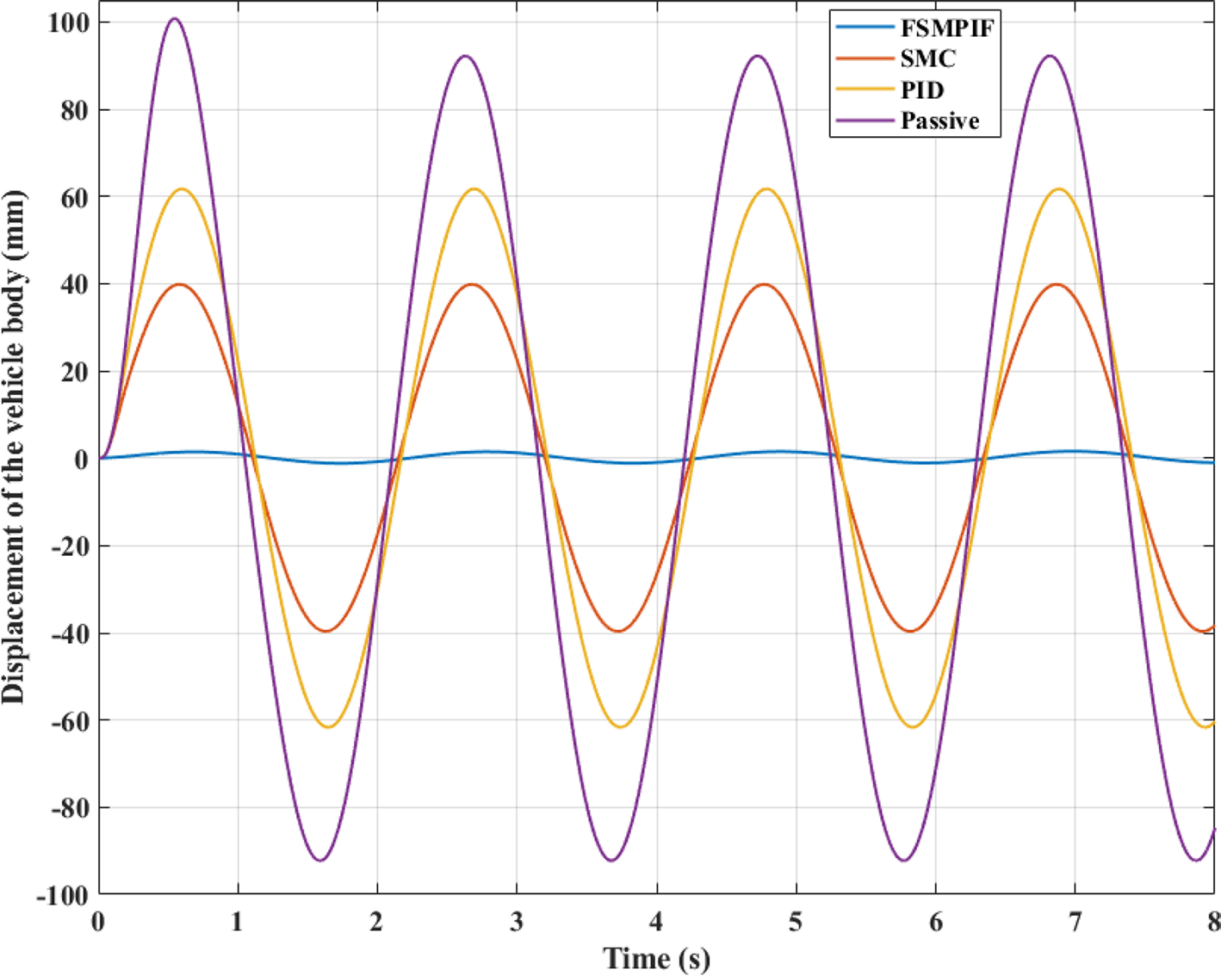
Displacement of the vehicle body (Case 1).

In the initial instance, a road surface excitation of the sine cyclic form is utilized. According to this rule, the displacement and acceleration of the vehicle body will cyclically vary. Figure 7 depicts the change in sprung mass displacement over time. If an automobile has a mechanical suspension system, its maximum displacement can reach 100.83 (mm). This value can be decreased by employing an active suspension system. This result indicates that the vehicle body displacement is only 61.72 (mm) and 39.84 (mm), respectively when the PID and SMC algorithms handle the active suspension system. In particular, once the FSMPIF algorithm is used to control an active hydraulic suspension system, the maximum displacement value may decrease drastically, reaching about 1.55 (mm). Compared to the initial circumstance, this is merely 1.54%. This is a highly positive outcome.

When evaluating vehicle stability, a mean vibration value must also be considered. This value may be determined using the RMS standard. According to simulated data, the sprung mass average displacement achieved 65.60 (mm), 43.60 (mm), 28.16 (mm), and 0.95 (mm) for the four examination scenarios. Using the value of the first scenario as a reference, the following numbers may be transformed equivalently to 100%, 66.46%, 42.93%, and 1.45%, respectively.

**Figure 8:**
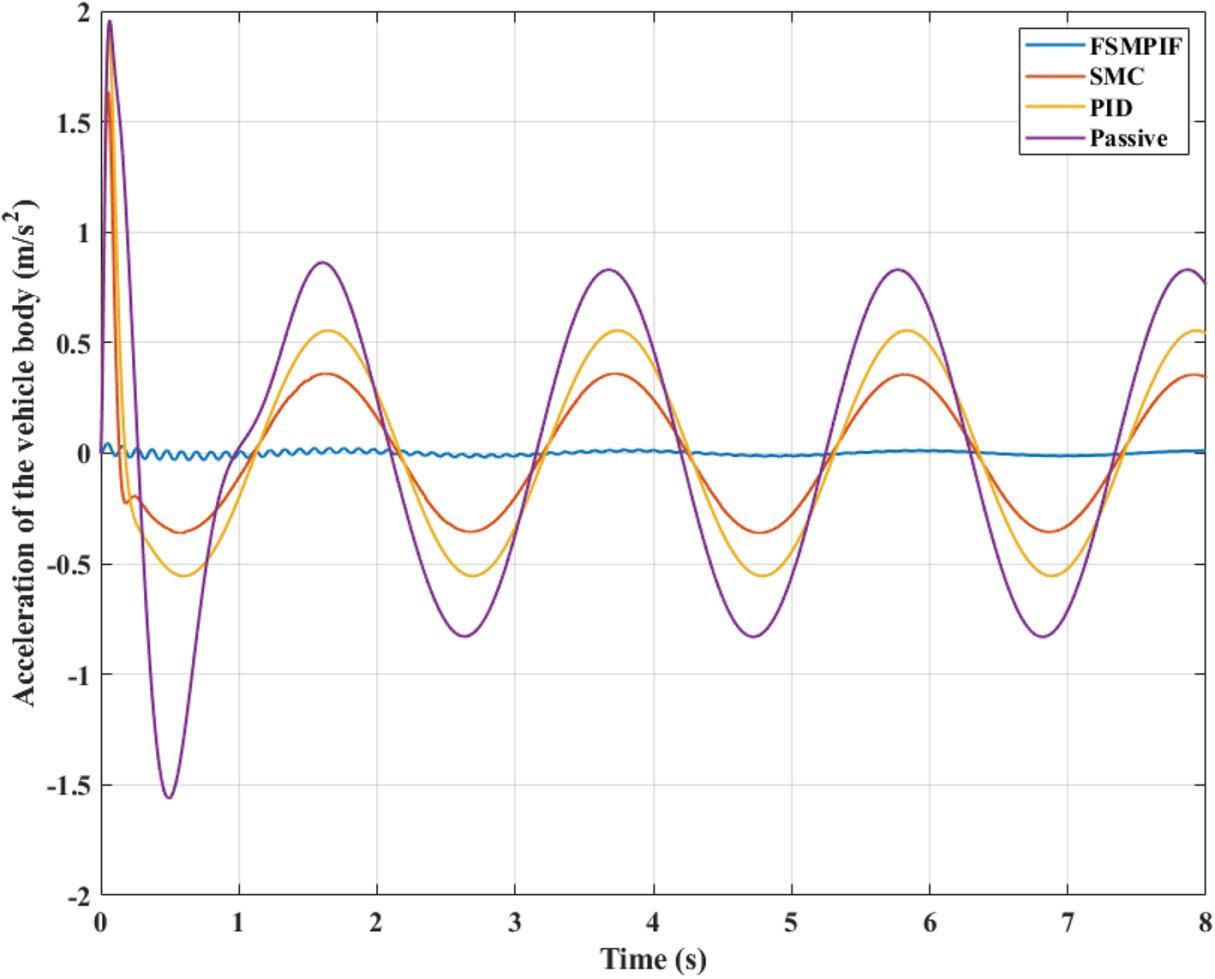
Acceleration of the vehicle body (Case 1).

The acceleration of a sprung mass may be used to evaluate its vibrations. The value of vertical acceleration could be examined in this work. Figure 8's graph reveals that the greatest vertical acceleration for four simulated conditions is 1.96 (m/s^2^), 1.91 (m/s^2^), 1.64 (m/s^2^), and 0.05 (m/s^2^), in that order. Due to the continuous character of this vibration, the average value may also be determined using the RMS criteria. An average vertical acceleration of an automobile with passive suspension can reach 0.67 (m/s^2^). This number may be dramatically lowered to as low as 0.01 (m/s^2^) when the FSMPIF algorithm-controlled active suspension is utilized. This discrepancy is huge. Thus, the vehicle's comfort and stability may be significantly enhanced.

Considering the change in the acceleration value in percent, it can be clearly seen that the average value of the acceleration when using the FSMPIF algorithm is only 1.49% compared to the situation of the car without the controller for the suspension system. In terms of the SMC situation and the PID scenario, these numbers reach 43.28% and 64.18%, respectively. Regarding using the maximum value in comparison, if the value of the Passive situation is fixed at 100%, the values of the other three scenarios are only 2.55%, 83.67%, and 97.45%. The difference between the FSMPIF and the Passive situation is very large, while the difference between the SMC and the PID with the Passive is not much. This further helps demonstrate the efficiency of the new algorithm proposed in this article.

**Figure 9:**
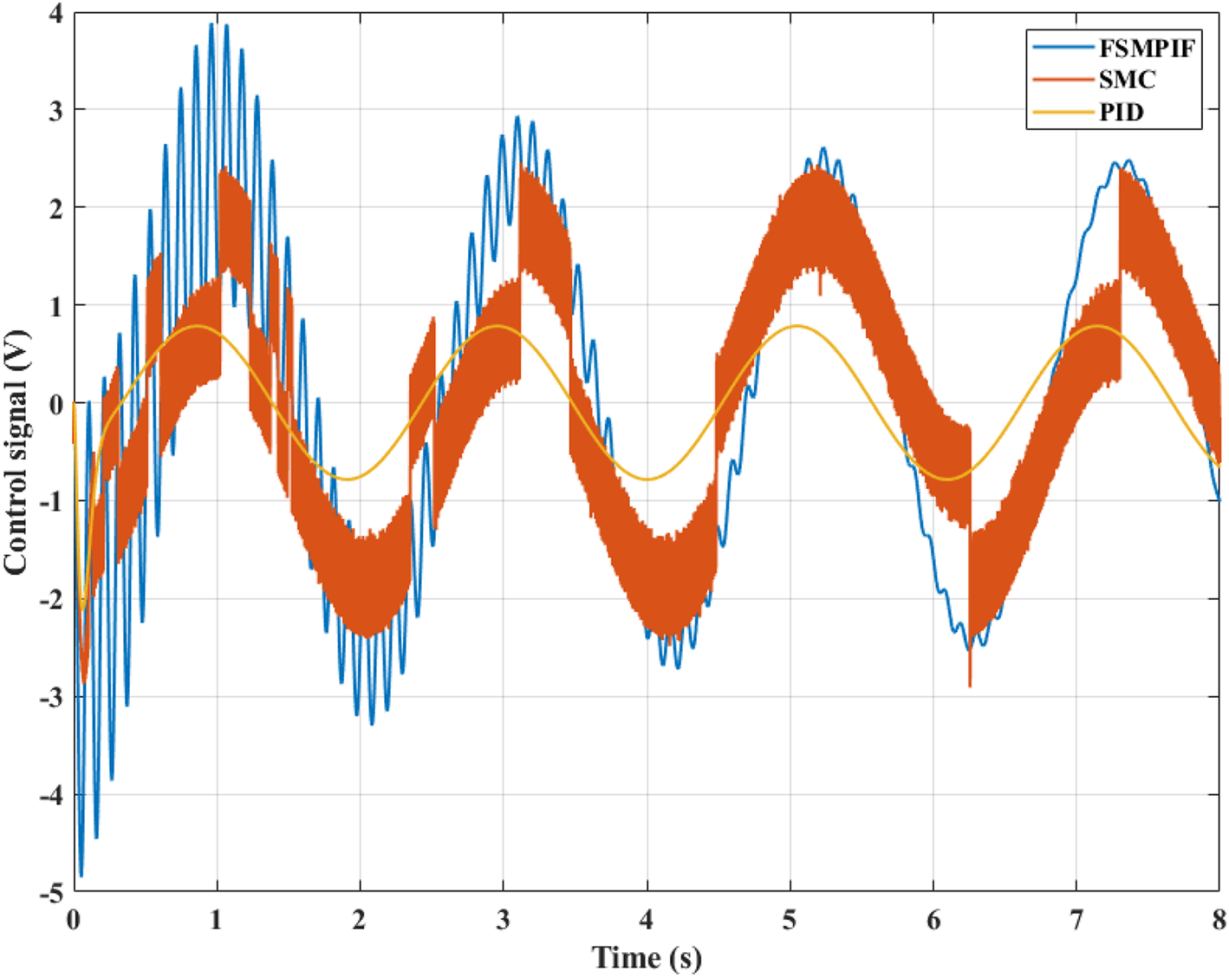
Control signal (Case 1).

The control signal for the system is shown in Fig. 9. According to this result, the voltage value in the FSMPIF situation is highest, but there is a decrease over time to return to a stable threshold. This is consistent with the car body acceleration result shown in Fig. 8. Meanwhile, the output signal of the conventional SMC controller is unstable, also known as the "chattering" phenomenon. The control signal of the PID algorithm is more stable, but its response is not good (it causes the car body to fluctuate more than SMC and FSMPIF).

#### Case 2

**Figure 10:**
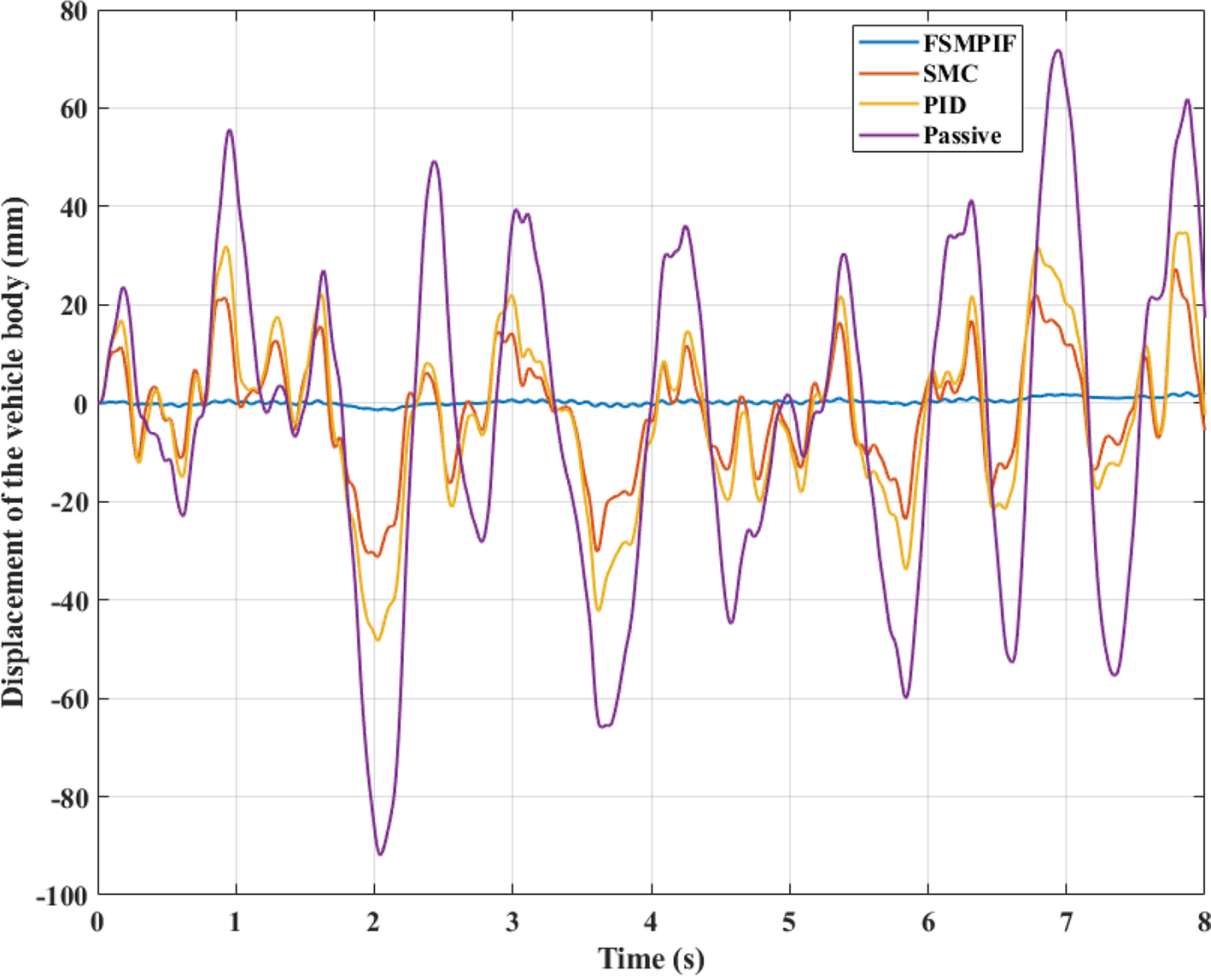
Displacement of the vehicle body (Case 2).

Random pavement stimulation is utilized in the second scenario. This is the actual variety of pavement. In this case, the amplitude and frequency of the vibrations are significantly greater than in the previous instance. Two results, including the vehicle body displacement and acceleration, are comparable to those in earlier scenarios. Additionally, the maximum and average values should be mentioned. The maximum permissible vehicle body displacement is 91.90 (mm) (Fig. 10). This number may be decreased by almost half to 48.29 (mm) if the PID algorithm handles the active suspension. This number can be decreased further by substituting the PID algorithm with the SMC method, which requires just 31.27 (mm). As soon as the new method FSMPIF presented in this article is used, the maximum displacement value may drastically decrease to 2.15 (mm). The average vibration values are 34.84 (mm), 17.48 (mm), 11.84 (mm), and 0.71 (mm). Up to 49.07 times, the difference between the readings is possible.

**Figure 11:**
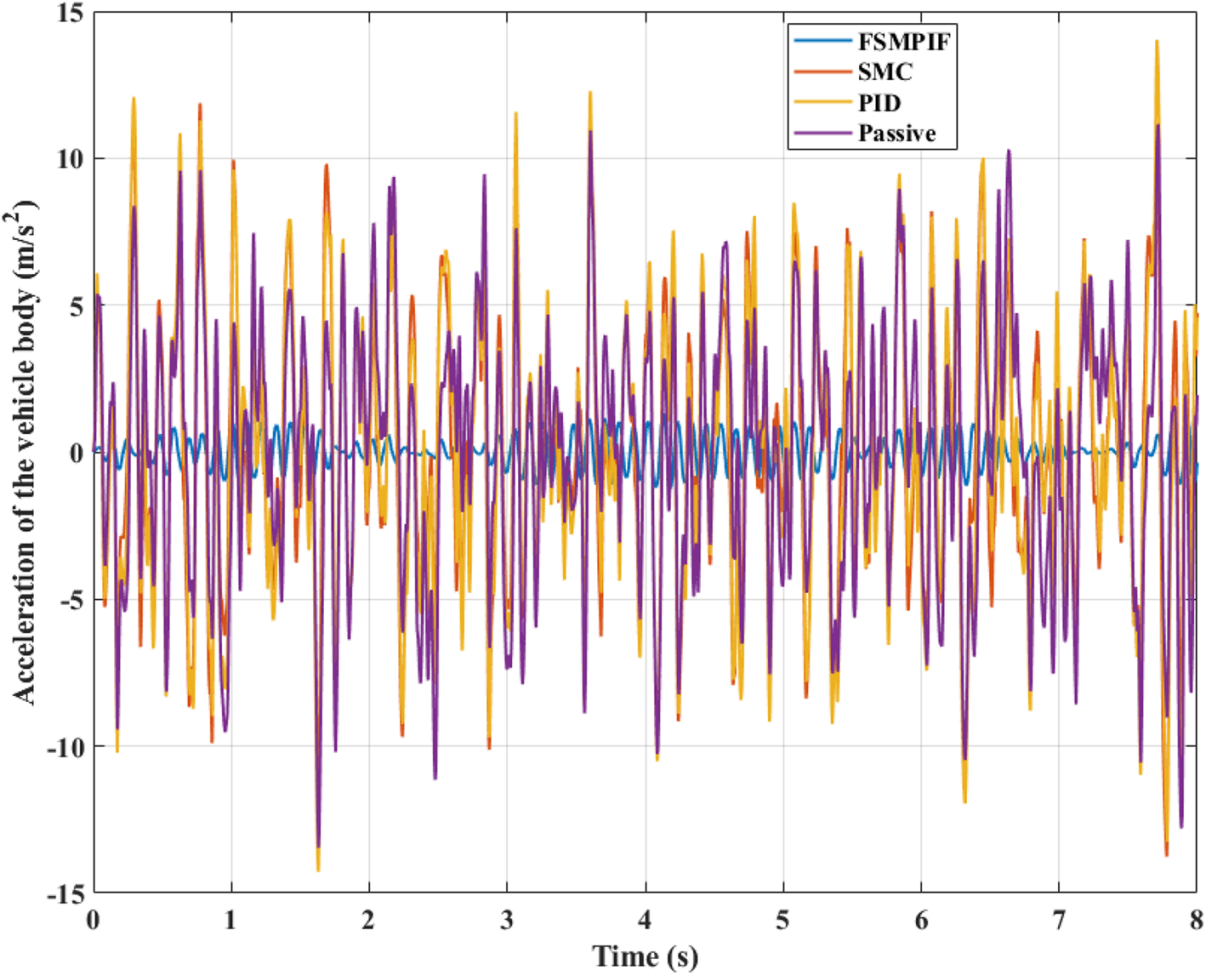
Acceleration of the vehicle body (Case 2).

In this situation, the acceleration value of the vehicle's body is relatively substantial. This might influence the vehicle's ride quality while in motion. These values regularly change throughout simulation time (Fig. 11). The maximum acceleration for an automobile with passive suspension is 13.45 (m/s^2^). If active suspension using SMC or PID algorithms is employed, the acceleration value can be more extensive. This affects the vehicle's comfort. Only after the FSMPIF algorithm is implemented will the vertical acceleration value drop. This decrease is significant, just about 1.30 (m/s^2^). For the two algorithms, SMC and PID, the percentage of maximum acceleration value are even more significant than that of Passive (102.30% and 106.17%, respectively). Meanwhile, the value belonging to FSMPIF is only 9.67%. In addition, the average values obtained from the calculation are 12.59%, 102.29%, and 108.24%, respectively, compared with Passive. Consequently, utilizing this innovative technique can increase vehicle stability.

The simulation's findings are presented in Table 3. The percentage differences between values are depicted in Table 4.

**Table 3:** Simulation results (value).

	FSMPIF		SMC		PID		Passive	
	Max	Ave	Max	Ave	Max	Ave	Max	Ave
Case 1								
Displacement (mm)	1.55	0.95	39.84	28.16	61.72	43.60	100.83	65.60
Acceleration (m/s^2^)	0.05	0.01	1.64	0.29	1.91	0.43	1.96	0.67
Case 2								
Displacement (mm)	2.15	0.71	31.27	11.84	48.29	17.48	91.90	34.84
Acceleration (m/s^2^)	1.30	0.55	13.76	4.47	14.28	4.73	13.45	4.37

**Table 4:** Simulation results (percent).

	FSMPIF		SMC		PID		Passive	
	Max	Ave	Max	Ave	Max	Ave	Max	Ave
Case 1								
Displacement (mm)	1.54	1.45	39.51	42.93	61.21	66.46	100	100
Acceleration (m/s^2^)	2.55	1.49	83.67	43.28	97.45	64.18	100	100
Case 2								
Displacement (mm)	2.34	2.04	34.03	33.98	52.55	50.17	100	100
Acceleration (m/s^2^)	9.67	12.59	102.30	102.29	106.17	108.24	100	100

**Figure 12:**
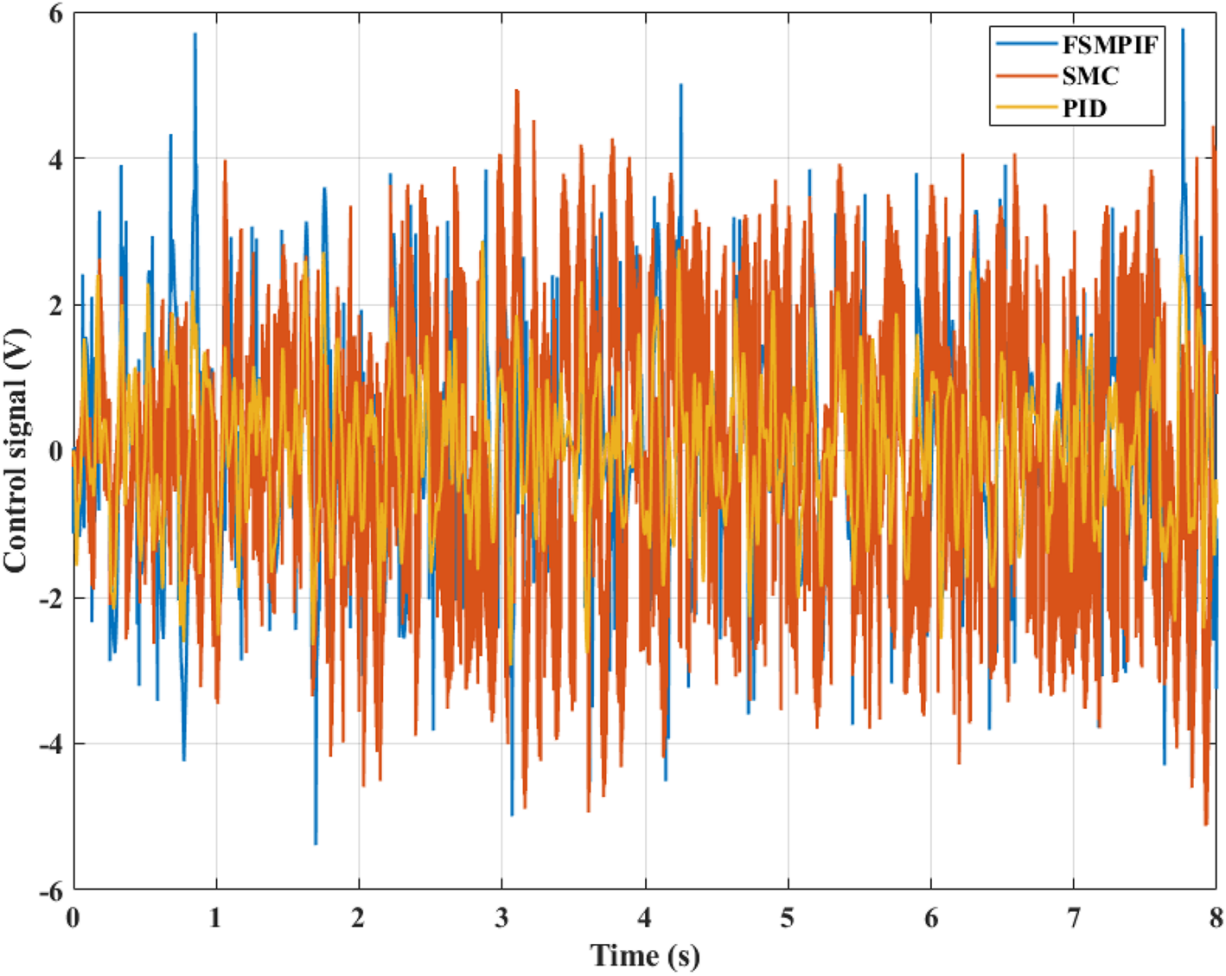
Control signal (case 2).

In the second case, the control signal changes continuously. The amplitude and frequency of the control signal are larger than in the first case (Fig. 12). The "chattering" phenomenon still occurs even when using only the traditional SMC algorithm. Meanwhile, the FSMPIF algorithm helps to limit this phenomenon more effectively.

## Conclusions

The roughness of the road surface can cause the vehicle's body to vibrate. This vibration will damage the passengers' riding comfort. Consequently, an active suspension system is utilized to address this issue. The controller of the active suspension system will have a significant impact on its performance. In this article, the FSMPIF active suspension control algorithm is described. The proposed algorithm by the author is strict. This method is a combination between intelligent control, linear control, and nonlinear control.

The displacement and acceleration data of the vehicle's body are used to determine vibration levels. Through numerical simulation, these values are determined. Simulation findings indicate that when the FSMPIF algorithm is employed to regulate the active suspension system, the car body's displacement and acceleration values are significantly decreased. In both instances under examination, the maximum and mean values of displacement and acceleration are small compared to other circumstances. As a result, the vehicle's smoothness and comfort have been improved. This new method yields positive results. This method, however, is rather complicated. So, it should be simplified in the future to be applied to automobile mechatronic systems. Further, vehicle vibration testing must be done to confirm the effectiveness of this new control mechanism.

## Data Availability

The datasets used and/or analysed during the current study available from the corresponding author on reasonable request.
